# Height and volume restoration in osteoporotic vertebral compression fractures: a biomechanical comparison of standard balloon kyphoplasty versus Tektona® in a cadaveric fracture model

**DOI:** 10.1186/s12891-020-03899-7

**Published:** 2021-01-13

**Authors:** Antonio Krüger, Martin Bäumlein, Tom Knauf, Hugues Pascal-Moussellard, Steffen Ruchholtz, Ludwig Oberkircher

**Affiliations:** 1 Department of Trauma Surgery, Orthopaedic Surgery and Spine Surgery, Asklepios Hospital Lich GmbH, Lich, Germany; 2 Center for Orthopaedics and Trauma Surgery, Philipps University Marburg, Baldingerstraße, 35043 Marburg, Germany; 3 Pitié- Salpêtrière Hospital, Université Pierre et Marie Curie - Paris VI, Orthopaedic dpt, Paris, France

**Keywords:** Vertebral body fracture, Osteoporosis, Compression fracture, Cement, Biomechanical

## Abstract

**Background:**

Standard balloon kyphoplasty represents a well-established treatment option for osteoporotic vertebral compression fractures. Aim of the present study was to evaluate two different methods of percutaneous augmentation (standard balloon kyphoplasty (BKP) versus Tektona® (TEK)) with respect to height restoration.

**Methods:**

Four-teen vertebral bodies of two female cadavers were examined. Fractures were created using a standardized protocol. CT-scans were taken before and after fracture, as well as after treatment. Afterwards two groups were randomly assigned in a matched pair design: 7 vertebral bodies (VB) were treated with BKP (Kyphon, Medtronic) and 7 vertebral bodies by TEK (Spineart, Switzerland) Anterior, central and posterior vertebral body heights were evaluated by CT-scans. Volumetry was performed using the CT-scans at three different timepoints.

**Results:**

Values before fracture represent 100%. The anterior height after fracture was reduced to 75.99 (± 4.8) % for the BKP group and to 76.54 (± 9.17) % in the TEK Group. Statistically there was no difference for the groups (*p* = 1). After treatment the values increased to 93.06 (± 5) % for the BKP Group and 87.71 (± 6.2) % for the TEK Group. The difference before and after treatment was significant for both groups (BKP *p* = 0.0006; TEK *p* = 0.03). Within the groups, there was no difference (*p *= 0.13).

The Volume of the vertebral body was reduced to 82.29 (± 8.4) % in the BKP Group and to 76.54 (± 8.6) % in the TEK Group. After treatment the volume was 89.26 (± 6.9) % for the BKP Group and 88.80 (± 8.7) % for the TEK Group. The difference before and after treatment was significant only for the TEK group (BKP *p *= 0.0728 n.s.; TEK *p* = 0.0175). Within the groups, there was no difference (*p* = 0.2).

The average cement volume used was 6.1 (range 3.6–9 ml) for the BKP group and 5.3 (3–7.2 ml) for the TEK group respectively.

**Conclusions:**

Based on our results the new System Tektona® in osteoporotic compression fractures might represent a promising alternative for the clinical setting, especially preserving bone. Further biomechanical tests and clinical studies have to proof Tektona®`s capabilities.

## Background

Standard balloon kyphoplasty represents a well-established treatment option for painful osteoporotic vertebral compression fractures [1]. An advantage compared to vertebroplasty is the potential of height restoration which was already described and named BAER (Balloon Assisted Endplate Reduction) [2, 3]. 50% of height restoration is achieved by prone position during surgery whereas another 50% can be related to inflation of the balloons [4]. Losing some of the height after deflation of the balloons is described and has been addressed by the development of several other techniques and devices [5–8]. Furthermore the inflation of the balloon follows the path of least resistance resulting in substantial damage of unbroken trabecular bone. Several alternative techniques with regard to height restoration and bone preservation have been emerged. Tektona® offers the theoretical advantage to allow the surgeon using the power of the device directly to where it is needed inside the vertebral body. Aim of the present study was to evaluate two different methods of percutaneous augmentation of vertebral compression fractures (standard balloon kyphoplasty (BKP) versus Tektona® (TEK)) with respect to height restoration in a biomechanical cadaver model.

## Methods

Two spines of two Caucasian females (73 and 81 years of age) were used (Source: Anatomy Gifts Registry, Hanover, MD, USA). The DXA-score confirmed osteoporosis (T-scores − 2.4 and − 3.7 respectively). The vertebral bodies from T8 to L4 were dissected and freed from surrounding tissues. Vertebral bodies with fractures and the corresponding vertebral body in the other spine were excluded. Standardized vertebral wedge compression fractures were created by a material testing machine (Instron® 5566) using an previously established fracture model [9]. In order to reduce the anterior height of the vertebral body an axial load was continuously increased until 30% of the initial height of the anterior endplate was reached. Compression force was maintained for 15 minutes. After the fracture two groups were randomly assigned in a matched pair design: 8 vertebral bodies (VB) were treated with balloon kyphoplasty (Kyphon, Medtronic) and 8 vertebral bodies by Tektona® (Spineart, Switzerland). CT-scans were taken before and after fracture, as well as after treatment.

### Operative technique

#### Balloon kyphoplasty

Two guidewires are placed bipedicular inside the vertebral body using Jamshidi-needles. Working cannulas are placed using the guidewires and the guidewires are removed. A bone drill is used to create space for two balloons which are expanded using a hydraulic device. Inflation of the balloon was stopped according to the surgeon’s preference. The balloons were removed and the cavities were filled with bone cement. Cementing was stopped on clinical judgement.

#### Tektona®

**Fig. 1:**
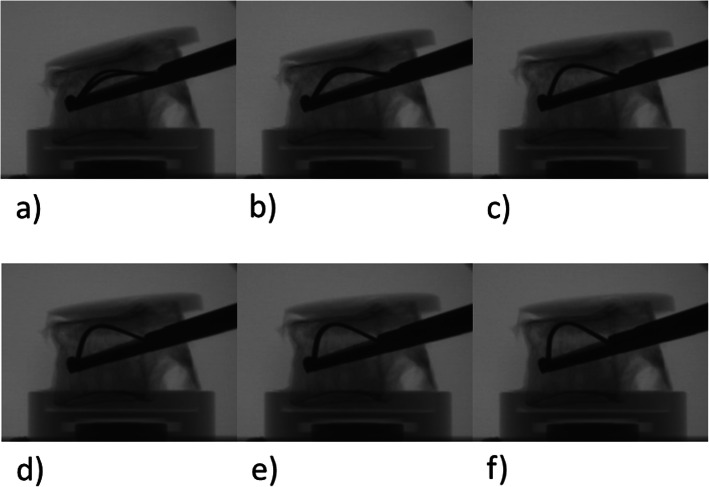
**a**-**f** Lateral views of the intraoperative setting. Sequential opening of the Tektona® blade. Notice the height restoration and uplifting of the upper endplate of the treated vertebral body): Lateral views of the intraoperative setting. Sequential opening of the Tektona® blade. Notice the height restoration and uplifting of the upper endplate of the treated vertebral body

Two guidewires are placed bipedicular inside the vertebral body using Jamshidi-needles. The working cannulas are mounted on a bone drill and are placed using the guidewires. The bone drill is used to create space for the two Tektona® devices. The drill and guidewires are removed afterwards. Tektona® consists of a lamella that can be sequentially expanded and retracted (Fig. 1a-f. This step can be performed repeatingly. The Lamella can be placed more anteriorly or posteriorly as well as turned around the axis of the device. The two lamellas were removed and the cavities were filled with bone cement. Cementing was stopped on clinical judgement.

### Measurements

The height restoration was measured via CT. Points of measuring were defined prior the study (sagittal midline of the vertebral body). Anterior, central and posterior height was measured in millimetres (mm) and then indicated in percent, with the non-fractured vertebral body representing 100%.

The vertebral body volume was measured via CT data. CT slices (0.7 mm, soft reconstruction kernel) were transferred to 3DSlicer 4.8 (www.slicer.org [10]). FastGrowCut plug in was used to segment the vertebral body and calculate its volume. Volume was measured in millilitres (ml) and then indicated in percent, with the non-fractured vertebral body representing 100%.

### Statistical analysis

The statistical analysis was performed using the GraphPad Prism Software Version 5.03 (GraphPad Software, Inc. USA). Due to the small group sizes, a non-parametrical test (Mann-Whitney-Test) was conducted. The significance level was set at *p* < 0.05.

## Results

One of the initial eight pairs (Level T9) of vertebral bodies was removed due to intraoperative technical problems (perforation of the endplates by the lamella, using too much force on the side of the surgeon). Alltogether 7 corresponding matched pairs of vertebral bodies were used. The absolute values measured in millimeters (mm) and milliliters (ml) before and after fracture as well as after treatment are listed in Table 1. Values before fracture represent 100%.

**Table 1:** Measurements of vertebral body heights (initial, fractured and cemented) for both methods (Absolut measures in millimeters (mm) and milliliters (ml))

Balloon Kyphoplasty vs. Tektona		
	**Balloon Kyphoplasty** *n* = 7	**Tektona** *n* = 7
	Mean and ± SD	Mean and ± SD
**Initial**		
Anterior Height (mm)	24.19 ± 3.76	23.84 ± 3.66
Central Height (mm)	21.20 ± 2.2	20.93 ± 2.53
Posterior Height (mm)	24.7 ± 2.29	24.91 ± 2.47
Vertebral Volume (ml)	24.33 ± 6.74	23.79 ± 5.52
**After fracturing**		
Anterior Height (mm)	18.33 ± 2.7	18.01 ± 1.76
Central Height (mm)	18.97 ± 2.41	17.81 ± 1.87
Posterior Height (mm)	24.66 ± 1.67	24.34 ± 2.12
Vertebral Volume (ml)	19.81 ± 5.06	17.99 ± 3.71
**After augmentation**		
Anterior Height (mm)	22.49 ± 3.58	20.97 ± 3.78
Central Height (mm)	20.66 ± 2.4	19.79 ± 2.43
Posterior Height (mm)	24.66 ± 1.68	25.01 ± 2.21
Vertebral Volume (ml)	21.60 ± 5.92	20.99 ± 4.69

The values of the percentages as well as the results of the statistical analysis (*p*-values) are listed in Table 2. The anterior height after fracture was reduced to 75.99 (± 4.8) % for the BKP group and to 76.54 (± 9.17) % in the TEK Group. Statistically there was no difference for the groups (*p* = 1). After treatment the values of the anterior height increased to 93.06 (± 5) % for the BKP Group and 87.71 (± 6.2) % for the TEK Group. The difference before and after treatment was significant for both groups (BKP *p *= 0.0006; TEK *p* = 0.03). Within the groups, there was no significant difference (*p* = 0.13).

**Table 2:** Measurements of vertebral body heights (initial, fractured and cemented) for both methods (percentages of the initial unfractured vertebel bodies (= 100%)

	Balloon Kyphoplasty *n* = 7	Tektona *n* = 7	
	Mean and ± SD	Mean and ± SD	Inbetween groups
**After fracturing**			
Anterior Height (%)	75.99 ± 4,77	76,54 ± 9.17	*p* = 1
Central Height (%)	88.78 ± 8.70	85.61 ± 8,25	*p* = 0.62
Posterior Height (%)	100.1 ± 4.03	97.85 ± 3.2	*p* = 0.3
Vertebral Volume (%)	82.29 ± 8.35	76.54 ± 8.56	
**After augmentation**			
Anterior Height (%)	93.06 ± 5.00 *p* = 0.0006	87.71 ± 6,2 *p* = 0.03	*p* = 0.13
Central Height (%)	96.65 ± 7.7 *p* = 0.097	94.56 ± 3.5 *p* = 0.0175	*p* = 0.9
Posterior Height (%)	100.0 ± 2.9 *p* = 1	100.5 ± 2.9 *p* = 0.12	*p* = 0.7
Vertebral Volume (%)	89.26 ± 6.93 *p* = 0.0728	88.80 ± 8.68 *p *= 0.0175	*p* = 0.2

The Volume of the vertebral body was reduced to 82.29 (± 8.4) % in the BKP Group and to 76.54 (± 8.6) % in the TEK Group. After treatment the volume was 89.26 (± 6.9) % for the BKP Group and 88.80 (± 8.7) % for the TEK Group. The difference before and after treatment was significant only for the TEK group (BKP p = 0.0728; TEK p = 0.0175). Within the groups, there was no significant difference (p = 0.2).

## Discussion

Balloon kyphoplasty was designed to improve patients safety by reducing the risk of cement leakage [11–13]. An additional advantage compared to vertebroplasty is the potential of height restoration in fresh fractures. The idea of endplate reduction using balloons was described several years ago and named BAER (Balloon Assisted Endplate Reduction) [2]. Voggenreiter [4] showed that 50% of height restoration achieved during surgery is based on patients positioning in prone position and that another 50% can be related to inflation of the balloons. Nevertheless some of the height that is gained during height restoration is lost after deflation of the balloons. This problem has been addressed by the development of several other techniques and devices [7–10]. Another disadvantage of the balloon is that the inflation can only be influenced by positioning of the uninflated balloon. The inflation of the balloon follows the path of least resistance. If in osteoporotic fractures height restoration is a treatment goal often substantial damage is caused to unbroken trabecular bone. Often the balloons have to touch the lateral walls of the vertebral bodies before craniocaudal expansion occurs. Interdigitation of cement with trabecular bone is limited [14]. The ideal tool for height restoration and endplate reconstruction would allow the surgeon to direct the power of the device directly to where it is needed inside the vertebral body. In addition removal or the possibility of repositioning if the goal is not achieved by the first attempt would be preferable. Tektona® offers the theoretical advantages to address all of the ideas mentioned above. The goal of this biomechanical test was to compare Tektona® to the gold standard of balloon kyphoplasty in an osteoporotic, biomechanical setting.

A standardized protocol was used to test both procedures. The creation of the fractures followed again a standardized protocol that has been used in several studies before [8, 14, 15]. Osteoporotic compression fracture with an anterior height restoration of 30% represent an accepted indication for treatment with balloon kyphoplasty after conservative treatment fails. Patients with acute traumatic fractures and underlying osteoporosis that have to be admitted to the hospital on account of immobilizing pain are often treated surgically in Germany in the first week of the hospital stay if the immobilizing pain cannot be controlled conservatively [16, 17]. Treatment goal in the elderly is early return to daily activities and to prevent prolonged immobilisation. Pain reduction and safety still remain the first priorities in the treatment. Additional theoretical advantages e.g. height restoration and endplate reduction of new devices may lead to improved patient’s outcome. As long as patients are not exposed to higher risks these treatments might be considered for well-designed clinical studies.

The creation of an osteoporotic wedge compression fracture with anterior height reduction of 30% without involvement of the posterior wall was the goal of the first step of the fracture protocol. The fractures that were created gave comparable conditions for both treatment groups (see Tables 1 and 2).

In this biomechanical setting both procedures worked safely. However in one case the endplate of the vertebral body was damaged. This was one of the first cases in the laboratory and the handling with the device in a smaller vertebral body (T9) was demanding. The very experienced surgeon and first author (AK) used too much force opening the lamella. This pitfall has to be considered surgeon related and not device related. In all other vertebral bodies both devices were used safely and without any further problems. In all percutaneous approaches and techniques preoperative planning and intraoperative imaging gain increasing importance. The size of the vertebral body, the diameter of the pedicle and the fracture morphology have to be carefully analysed before surgical treatment.

**Fig. 2:**
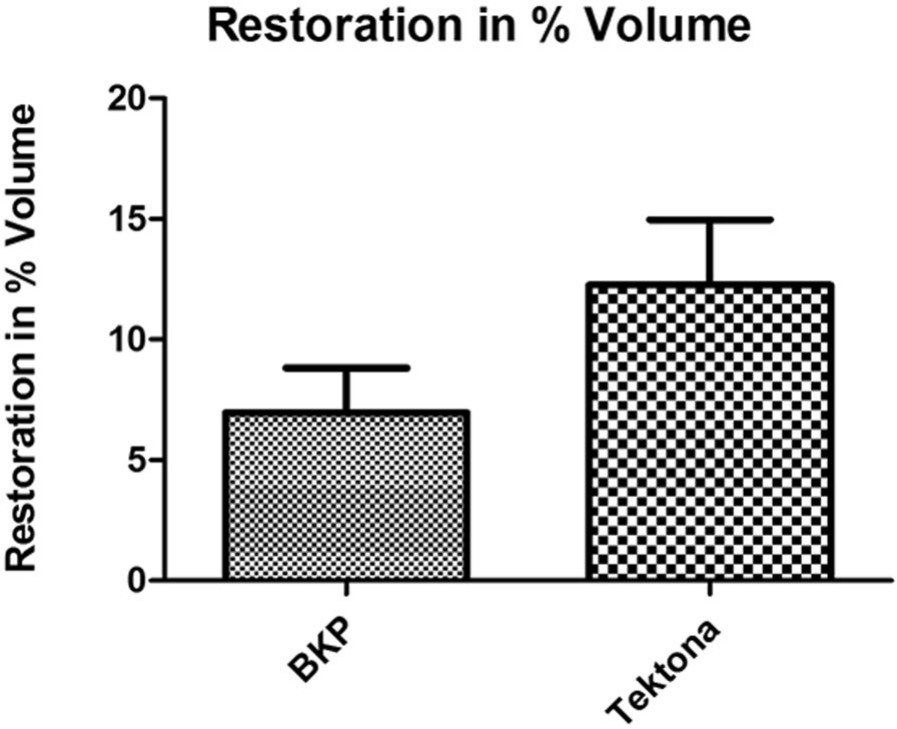
Illustration of the restoration of the vertebral volume given as percentage of the initial vertebral volume

After treatment statistical significant improvements for both groups regarding anterior height restoration were witnessed. The differences in between the both groups were not significant. The treatment with Tektona® resulted in a significant restoration of the central height. For BKP the difference was not significant. The differences in between the both groups regarding central height restoration was not significant. Additional vertebral body volume measurement was performed in order to achieve better visualization of the complete vertebral body restoration. Compression fractures will lead to vertebral body volume reduction due to trabecular bone destruction. Vertebral body volume measurements before fracture, after fracture and after treatment may show restoration of the vertebral body by surgical treatment. The treatment with Tektona® resulted in a significant restoration of the volume of the vertebral body (Fig. 2). For BKP the difference was not significant. The differences in between the both groups regarding volume restoration was not significant. These results show that the use of Tektona® leads to almost similar results when compared to the gold standard of balloon kyphoplasty. For central height restoration as well as for volume restoration Tektona® showed a trend to better results. Statistically there was no difference in between the groups.

Another potential advantage might be, that less intravertebral trabecular structures are destroyed using Tektona® compared to a balloon. This might lead to a more stable outcome and less refractures or collapse of the treated vertebrae.

## Conclusions

A study using cadaveric vertebrae was used to examine the height restoration of two different augmentation procedures used to treat osteoporotic vertebral compression fractures. The protocols for creating wedge fractures led to reproducible results and effects. The study showed that anterior and central as well as volume restoration was significantly improved with both techniques. Tektona® showed that it leads to comparable results. The power can directly be applied to the endplates, leaving more trabecular bone intact.

The clinical implications include might lead to an improved clinical outcome and biological healing process. Additional studies with different fracture types, cyclic loading and different bone qualities will help us understand this better.

## Data Availability

The datasets used and analyzed during the current study are available from the corresponding author on reasonable request.

## Abbreviations

BKP: Balloon Kyphoplasty (Kyphon, Medtronic)
TEK: Tektona® (Spineart, Swizerland)
VB: Vertebral body
CT: Computed tomography
